# HOMCOS: an updated server to search and model complex 3D structures

**DOI:** 10.1007/s10969-016-9208-y

**Published:** 2016-08-13

**Authors:** Takeshi Kawabata

**Affiliations:** Institute for Protein Research, Osaka University, 3-2 Yamadaoka, Suita, Osaka 565-0871 Japan

**Keywords:** Template-based modeling, Complex, BLAST, KCOMBU

## Abstract

The HOMCOS server (http://homcos.pdbj.org) was updated for both searching and modeling the 3D complexes for all molecules in the PDB. As compared to the previous HOMCOS server, the current server targets all of the molecules in the PDB including proteins, nucleic acids, small compounds and metal ions. Their binding relationships are stored in the database. Five services are available for users. For the services “Modeling a Homo Protein Multimer” and “Modeling a Hetero Protein Multimer”, a user can input one or two proteins as the queries, while for the service “Protein-Compound Complex”, a user can input one chemical compound and one protein. The server searches similar molecules by BLAST and KCOMBU. Based on each similar complex found, a simple sequence-replaced model is quickly generated by replacing the residue names and numbers with those of the query protein. A target compound is flexibly superimposed onto the template compound using the program *fkcombu*. If monomeric 3D structures are input as the query, then template-based docking can be performed. For the service “Searching Contact Molecules for a Query Protein”, a user inputs one protein sequence as the query, and then the server searches for its homologous proteins in PDB and summarizes their contacting molecules as the predicted contacting molecules. The results are summarized in “Summary Bars” or “Site Table”display. The latter shows the results as a one-site-one-row table, which is useful for annotating the effects of mutations. The service “Searching Contact Molecules for a Query Compound” is also available.

## Introduction

Molecular interactions are the essence of molecular functions in all forms of life, and thus characterizing interacting molecular pairs and interaction sites is fundamental for molecular biology. Huge numbers of interacting protein–protein pairs, and compound-protein pairs have been analyzed and stored in databases [1, 2]. The 3D structures of molecular complexes reveal the atomic details of molecular interactions, and thus provide important information to understand the molecular mechanisms [3]. The amount of 3D complex structural data in the PDB increased rapidly; however, it is still much less than that of reported interactions [4]. To fill this gap, computer modeling approaches are frequently performed to extend the data of 3D complex structures. Both template-based modeling and *de novo* modeling are performed to model 3D complex structures. Usually, template-based modeling is performed first, because it has advantages in terms of accuracy and computation costs, if proper templates are available.

Various methods for template-based modeling have been proposed. They can be roughly classified into two categories: “complex threading” and “template-based docking”. Complex threading is modeling from two or more amino acid sequences, and these sequences are aligned (threaded) on the template structure. Szilazyi and Zhang further classified complex threading into two subcategories: “monomer threading and oligomer mapping” and “dimeric threading” [5]. The “monomer threading and oligomer mapping” approach is a simple extension of the standard template-based modeling (homology modeling) of a monomeric structure. For each target input sequence, its template structures are searched by the sequence search methods [6–11] or the monomeric threading methods [12, 13]. If a template structure found for one target sequence forms a complex with that for another target sequence in the biological units in the PDB, then they can be regarded as the template 3D structure of the target sequences. The fitness of the target sequences and the template structure is evaluated by the sequence similarities or the potential energies between protein chains [6–10]. In the “dimeric threading” approach, two target sequence are simultaneously aligned with two corresponding structures using the two-body protein–protein interfacial energies [13–16].

The “template-based docking” method basically requires two or more monomeric 3D structures of the target proteins as inputs. For each target input structure, its similar structures are searched in the PDB. If a template structure found for one target structure forms a complex with that for another target structure in the biological units in PDB, then the target structures are superimposed on the corresponding template structure to generate a complex 3D model of the target structures. The reason why this method is called “template-based docking”, is that the standard de novo docking program also requires two monomeric protein 3D structures. The modeled 3D structure can be used instead of experimentally obtained 3D structures. To enhance the coverage of the prediction, most methods employ the monomer 3D modeling calculation as their first step [17–21]. The searches for the template structure are performed using various methods, including sequence similarity search, global 3D structure similarity search [17–21], and local 3D structure similarity search among interfaces [22, 23]. Since 3D structural searches, and especially local 3D structural searches can capture remote structural homologies and analogies, they can cover vast amounts of known interactions, although their accuracies are limited [24, 25].

Template-based modeling has also been applied to the modeling of small compound-protein complexes. For this approach, a target chemical compound is superimposed onto the template compound using the 3D structural alignment programs of chemical compounds [26]. The standard chemical compound—protein docking calculations are often improved by using known compound-protein complexes as the templates [27–30]. For most of these cases, the 3D structure of the target protein is assumed to be known, and only that of the target compound is predicted. Complex 3D models of both the compound and protein have also been built by template-based modeling approach [31, 32].

Nucleotide-protein 3D complex structures can been modeled using the template-based approach. To determine the target sites of DNA-binding proteins, the DNA sequences within protein-DNA complexes were replaced and the interaction energies were evaluated [33]. Recently, template-based methods for modeling both DNA/RNA and proteins were proposed [34–37].

Many WEB servers for modeling complex structures have been developed. Most of their targets are hetero protein dimers [7, 8, 10–12, 22, 23, 38], although a few servers are available for compound-protein and nucleotide-compound complexes. To annotate protein’s function completely, information about all types of protein complexes is required, because proteins often bind many types of molecules, such as other proteins, small compounds, metals, and nucleotides, to perform their functions.

Our server HOMCOS (HOmology Modeling of Complex Structure; http://homcos.pdbj.org) keeps the name of the server established in 2008 (http://strcomp.protein.osaka-u.ac.jp/homcos) [9]. However, we have completely rebuilt the server from scratch, and the current HOMCOS server is very different and much more useful than the previous one. The previous HOMCOS server only handled protein–protein 3D complexes. In contrast, the current server contains all of the molecules in the PDB, including proteins, nucleic acids, small compounds and metal ions. The new server is able to perform both the “complex threading” and “template-based docking” approaches, since it accepts both amino acid sequence and monomeric 3D structure as queries. For compound-protein modeling, the 3D structure of a chemical compound can be used as the query. HOMCOS employs the standard program BLAST for finding protein templates [39], and the program KCOMBU for finding compound templates [30, 40, 41]. Another new and useful service is the ability to search for contact molecules for a query protein. For a user-input query sequence, HOMCOS searches its homologous proteins in the PDB, and summarizes their contacting molecules as the predicted contact molecules. The results can be summarized as a table for each site of the query protein. This is useful for annotating functional effects of nsSNP.

## Materials and methods

### Data architecture of the HOMCOS database system

**Fig. 1:**
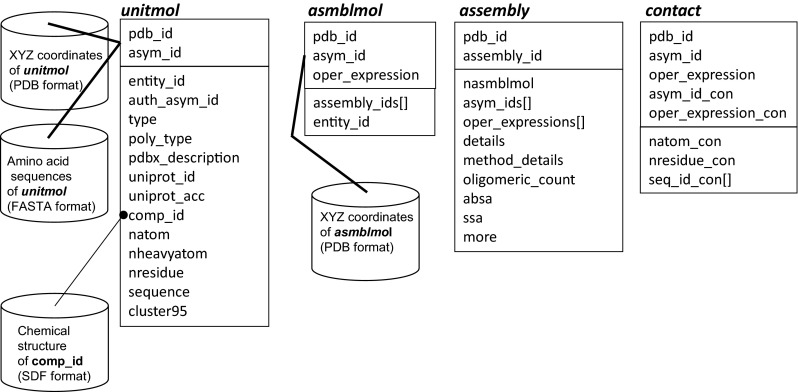
Diagram of the HOMCOS database system. *Boxes* labeled *unitmol*, *asmblmol*, *assembly*, and *contact* represent tables of the relational database. Each table is composed of two boxes: an *upper box* and a *lower box*. Attributes in the *upper box* are the primary key of the table. An attribute with brackets [] represents that its data type is an array, such as assembly_ids[] in the table *asmblmol*. Each *cylinder* represents are a set of files stored in the server

**Fig. 2:**
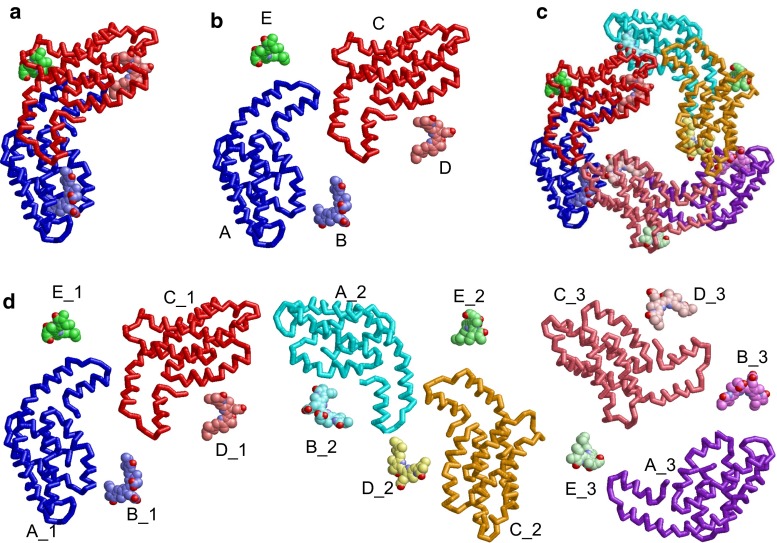
Molecular data structure of the HOMCOS system. Phycocyanin from *Sinechocystis* sp.PCC 6803 (PDBcode:4f0t) is used as an example. **a** 3D structure of the asymmetric unit. **b** 3D structures of five *unitmol* molecules. The labels “*A*”, “*B*”, “*C*” are asym_id indices. **c** 3D structures of the biological unit with assembly_id = 1. **d** 3D structures of 15 *asmblmol* molecules, which compose the biological unit with assembly_id = 1. The labels such as “*A_1*”, “*B_1*”, “*D_1*”, “*D_2*”,… are combinations of their asym_id and oper_expression. To visualize all the molecule separately, the molecule in **b**, **c** are translated from their original positions

The HOMCOS database system extracts 3D structural information from mmCIF files of the PDB [42], and stores them in a relational database (RDB). The 3D structure of the complex is mainly represented by four tables: *unitmol*, *asmblmol*, *assembly* and *contact*, as shown in Figs. 1 and 2.

The *unitmol* table describes the molecule in the asymmetric unit. Its primary key is a combination of pdb_id and asym_id. The asym_id is a new molecular identifier introduced in the mmCIF format, is often written as a capital letter (‘A’, ‘B’, ‘C’…). The classical PDB format also has a chain identifier, which is uniquely assigned to each protein and nucleotide polymer. In contrast, the new asym_id is assigned for all of the molecules, except waters, in the asymmetric unit (Fig. 2b). Therefore, all of the molecules in the PDB, not only proteins, but also nucleotides, small compounds and metals, have the corresponding *unitmol* data. The 3D coordinates of atoms in the *unitmol* are separately stored in the server, using the classical PDB format file. Their DSSP files are also stored, to retain their secondary structures and solvent accessibilities [43]. The amino acid sequences of the *unitmol* molecules are stored in a FASTA format file for the BLAST search [39]. Note that the residue numbers of the stored PDB files are mmCIF’s label_seq_id, not auth_seq_id; therefore, the residue numbers of the PDB files are consistent with the residue numbers of the FASTA file sequences in our server. For the *unitmol* molecules with a three-letter code (comp_id), their SDF files are stored in the server for the chemical structure search. It does not include any multi-residue molecules, such as peptides and oligosaccharides.

To summarize the predictions performed by the HOMCOS server, the redundancies of the sequences must be reduced. We perform an all-vs-all *blastp* comparison among all of the amino acid sequences of the *unitmol* molecules [39], and execute the single linkage clustering for them with sequence identity ≥95 % and coverage of aligned region ≥80 %. The label for the cluster is registered in the attribute cluster95 in the table for each protein *unitmol* molecule.

The *asmblmol* describes the 3D structure of the molecule in the biological unit. The mmCIF file contains information about the biological unit provided by both the authors and the software. Some of the biological unit are constructed by combining transformed *unitmol* molecules as shown in Fig. 2c. In the mmCIF file, the oper_expression identifier is assigned for the operation (rotation and translation) required to construct the biological units. Most of the oper_expression identifiers are numbers (‘1’, ‘2’, ‘3’,…), although some virus entries have special strings “PAU” and “XAU”, and some entries have a combination of two operations, such as “1-61” and “5-62” (PDB code:1m4x). The primary key of *asmblmol* is the combination of three ids, pdb_id, asym_id and oper_expression. The XYZ coordinates of the *asmblmol* molecule are also separately stored in a classical PDB format file. By definition, the amino acid sequence and the 2D chemical structure of each *asmblmol* molecule are the same as those of the corresponding *unitmol* molecule.

The table *assembly* summarizes the information about an assembly (biological unit), which is composed of several *asmblmol* molecules. Its primary key is a combination of pdb_id and assembly_id. A list of the *asmblmol* molecules of the assembly is stored in two array variables, asym_ids[] and oper_expressions[]. Note that many PDB entries have more than one candidate of their biological unit. For example, the PDB entry 3gyr has eight biological unit candidates; two homo hexamers (assembly_id = 1 and 2) defined by the author, and six homo dimers (assembly_id = 3, 4, …, 8) defined by the softwares PISA and PQS. Since there is no reliable standard for choosing one correct biological unit, the HOMCOS server contains all of the biological units described in the mmCIF file.

The table *contact* contains contacting residues between two *asmblmol* molecules belonging to the same biological unit (*assembly* data). If the distance between one of the heavy atom pairs of the two molecules is within 4 Å, then these two molecules are regarded as contacting molecule pairs and registered in the *contact* table. Its primary key is the combination of five keys: pdb_id, and one *asmblmol* key (asym_id, oper_expression) and another *asmblmol* key (asym_id_con, oper_expression_con). The array variable seq_id_con[] contains the residue numbers (seq_id) of the residues in the *asmblmol* molecule (asym_id, oper_expression) contacting with another *asmblmol* molecule (asym_id_con, oper_expression_con).

### Outline of the HOMCOS server

**Fig. 3:**
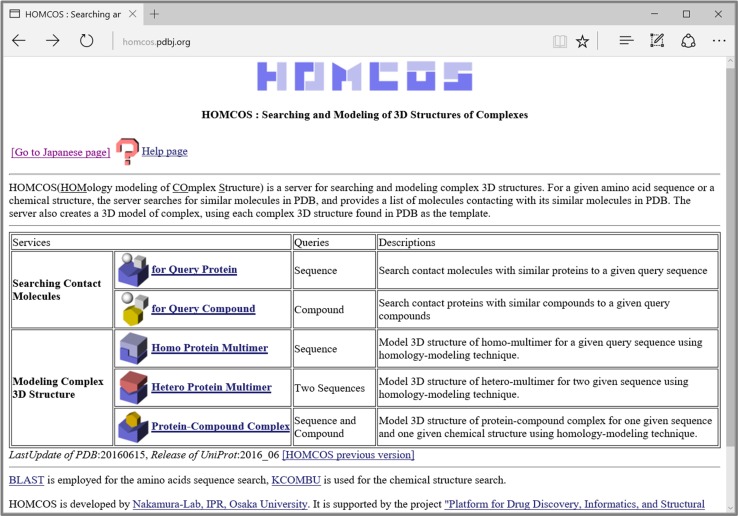
The top page of the HOMCOS server. Five services are provided

The top page of the HOMCOS server (http://homcos.pdbj.org) is shown in Fig. 3. The services are roughly classified into two categories: “searching contact molecules” and “modeling complex 3D structure”. The former contains two services: “for query protein” and “for query compound”. The latter contains three services: “homo protein multimer”, “hetero protein multimer”, and “protein-compound complex”. All five of the services are based on the common database system described in the previous subsection, and employ the 3D model view page, as explained below. In the following subsections, we will explain these services one by one, except for “homo protein multimer”, which is similar to “hetero protein multimer”.

### Searching contact molecules for a query protein

**Fig. 4:**
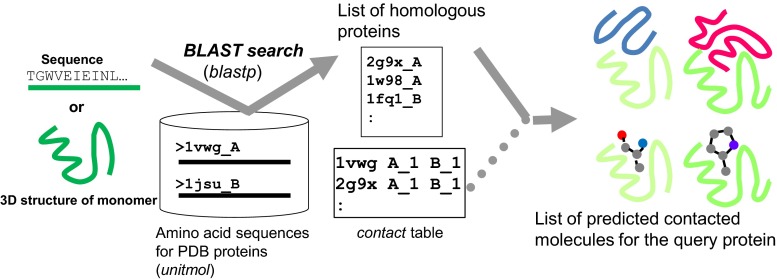
Overview of the service “searching contact molecules for query protein”

We first introduce the service for searching contact molecules for a query protein. An overview of the service is shown in Fig. 4. A user can specify a single query protein by various methods: an amino acid sequence, UniProt ID, PDB_ID + CHAIN_ID, and uploading a PDB file. The server performs a *blastp* search [39] with the given query protein sequence for all of the *unitmol* sequences in the PDB, to make the list of the homologous proteins (*unitmol*). The molecules contacting these homologous *unitmol* proteins are obtained by searching the *contact* table, and they are regarded as predicted contacting molecules with the query protein.

**Fig. 5:**
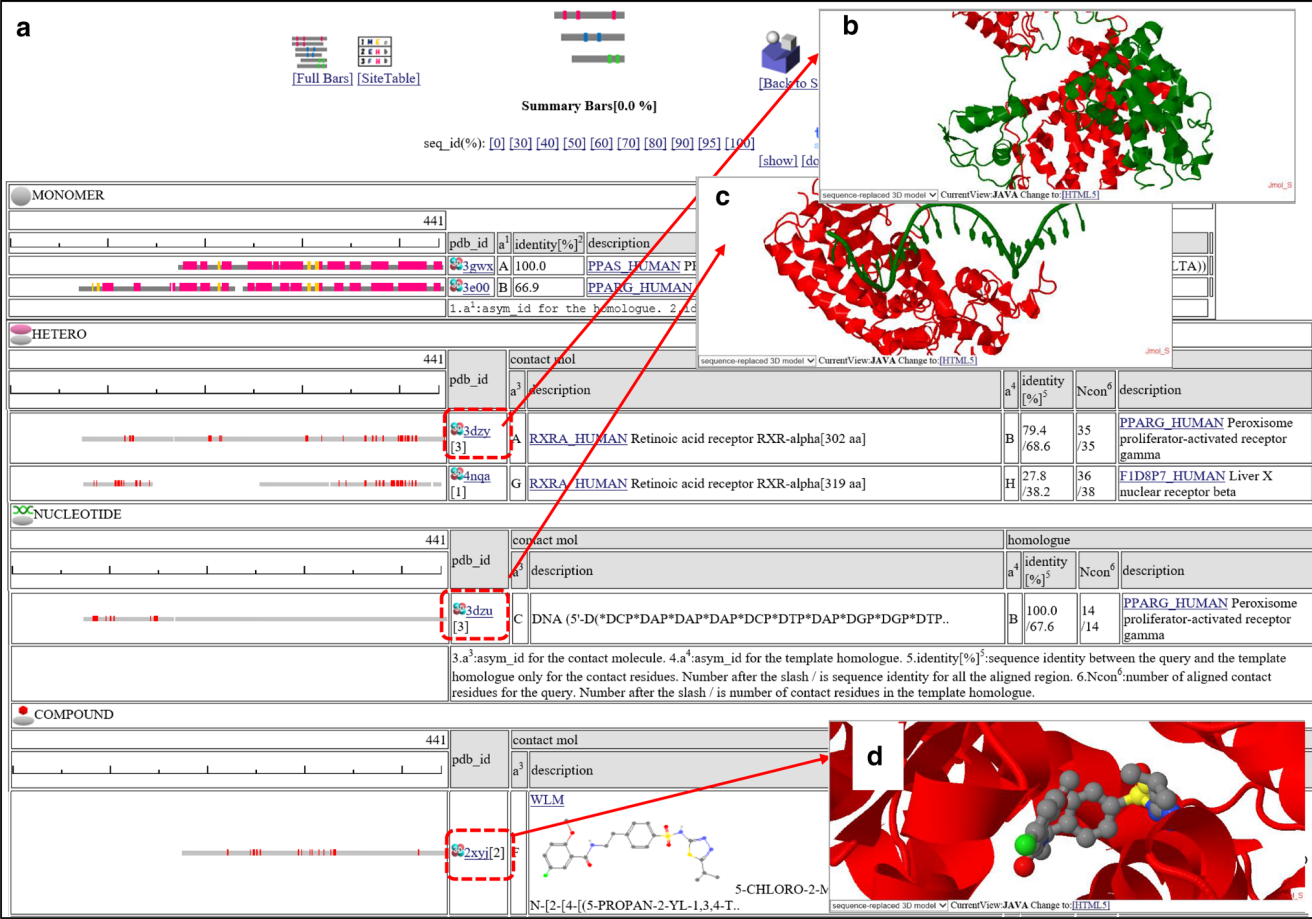
Snapshots of the contact “Summary Bars” view of “searching contact molecules for query protein”. The amino acid sequence of human PPAR-delta (PPARD_HUMAN) is used as the query. **a** The Summary Bars view. Bars show regions of aligned homologous structure. The width of the bar corresponds to the length of the query protein. In the top “MONOMER” table, the secondary structures are shown by colored bars (*red*: *α*-helices, *yellow*: *β*-strands). In the other tables (“HETERO”, “NUCLEOTIDE”, and “COMPOUND”), contacting residues with other molecules are shown in small red boxes. If a user clicks the “3D” icon, then the 3D model view windows appears. In the column “identity[%]”, two sequence identities are shown, such as “79.4/68.6” and “100.0/67.6”. The first number is the sequence identity of the contact site, and the second number is the identity of all the aligned sites. The identity of the contact site is a good measure for the quality of the model, especially for the small chemical compound. **b** A 3D model of a hetero protein complex, based on the template 3dzy. The contact protein is RXRA_HUMAN (asym_id = A), and the homologue is PPARG_HUMAN (asym_id = B; sequence identity = 68.6 %). **c** A 3D model of a protein-nucleotide complex based on the template 3dzu. The contact molecule is the DNA single strand (asym_id = C). The homologue is PPARG_HUMAN (asym_id = B; sequence identity = 67.6 %). The figure shows one protein – single stranded DNA complex; however, the biological unit of 3e00 is composed of two protein chains and double stranded DNA. To display all of the molecules in the biological unit, click the icon “ALLMOL” in the 3D model view page (see Figs. 11, 12). **d** A 3D model of a protein-compound complex based on the template 2xyj. The contact molecule is WLM (asym_id = F). The homologue is PPARD_HUMAN (asym_id = A; sequence identity = 100 %). This complex is an experimentally determined 3D structure for the query complex, rather than a prediction

These results can be summarized in two ways: Summary Bars and Site Table. The Summary Bars view shows the information about the predicted 3D complexes in a colored-bar format. An example of the Summary Bars view is shown in Fig. 5. The width of the bar corresponds to the length of the query protein. Aligned regions are shown in gray-bars, and residues contacting the other molecule are shown in small red boxes. At the top of the page, the UniProt feature tables [44] and monomeric 3D structures are shown. The predicted 3D complexes are classified into seven classes: hetero oligomers, homo oligomers, nucleotide-complexes, other polymer-complexes, small compound complexes, metal complexes, and precipitant complexes. If a user clicks the “3D” icon, then the 3D structure of the complex is shown in the 3D model view page. From the 3D model view page, a user can download the 3D model structures with a sequence-replaced query structure and other binding molecules. The details will be described in the subsection “3D model viewer”.

Some protein families, such as globin and protein kinase, have hundreds of 3D structures in the PDB with various ligands and different mutations. Too many homologues yield too many bars on the page, which makes it very complicated for users. To solve this problem, the representative bars are shown in the default setting. For the monomer 3D bars, homologous monomeric 3D structures are listed in the order of increasing *blastp* E-value, only if the number of additionally aligned residues >10. For the 3D complex bars, a single linkage clustering is performed for all of the homologous dimers in the same interaction class. Only one representative dimer is shown for each cluster. The criteria of linking are as follows: (1) “type” of contact molecule is identical. (2) Tanimoto coefficient of binding sites is more than 0.2. The “type” of the contact molecule is set to cluster95 for proteins (see the previous section about data architecture), the three-letter code (comp_id) for compounds, metals, precipitants, and sequence for nucleotide, and the pdbx_description for others (see attribute names in Fig. 1). The default setting of the service is the summarized “Summary Bars” page. If a user clicks the “Full Bars” icon, all of the predictions are shown on the page.

**Fig. 6:**
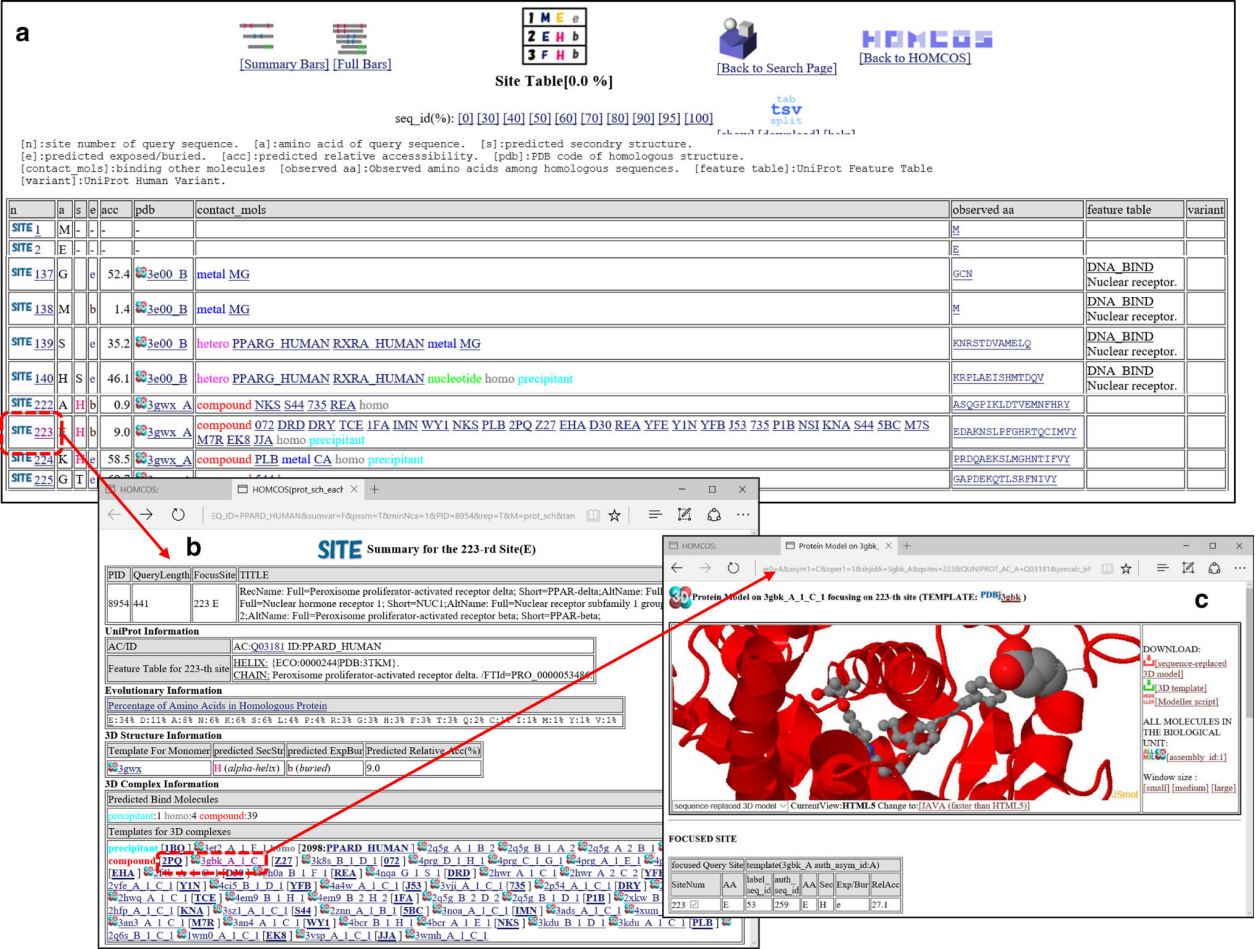
Snapshots of the “Site Table” view of “searching contact molecules for query protein”. The amino acid sequence of human PPAR-delta (PPARD_HUMAN) is used as the query. **a** Site table. Each *row* corresponds to each site of the query protein. **b** A window for the 223-rd site. **c** 3D model view for the complex 3D structure with the small compound 2PQ using the structure 3gbk as the template

Another view is called the “site table” view, which shows the information about the predicted 3D complexes in the “one site for one row” table format. This table is designed for analyzing the effects of amino acid mutations on the structure and the function. An example of the Site Table view is shown in Fig. 6a. Each row corresponds to one of the sites of the amino acid sequence, and it summarizes all of the information about the site: contacting molecules of homologues with the site, secondary structure and accessibility of the most similar structure, UniProt feature table and humsavar.txt [44]. Among the 3D structural features, accessibility is reportedly the most important to predict disease-associated mutations, as mutations in buried sites in a protein structure are more likely to lead to disease [45, 46]. The relationships between protein–protein interaction sites and disease-associated mutations are also reported [47–49]. Additionally, observed amino acid frequencies are shown, which are obtained from PSI-BLAST for the UniProt database [39]. These frequencies are essential to predict important functional sites, and mutational effects on the phenotype [50]. If a user clicks the “SITE” icon of each site, then the summary of the specific site appears (Fig. 6b), and if the “3D” icon is clicked, then the 3D model view of the complex 3D structure will appear with the highlighted specific site (Fig. 6c).

This service assumes that proteins with similar sequences should have similar binding properties. In other words, a query protein may bind to contact molecules of similar proteins to the query. Fukuhara et al. [9] reported that sequence similarity is the most effective feature to predict interacting protein pairs; however, its accuracies are limited for remote homologues. Users have to be careful when interpreting our server’s predictions, especially with those based on weak similarities. On the top of the page in Figs. 5a and 6a, the links to change the threshold value of sequence identities (seq_id(%)) from 0 to 100 %. The default is 0 %, which means that only *E*-value <0.0001 is the criterion to choose the homologues. If the users would like to focus on more reliable predictions, we recommend an increase in the sequence identity.

### Searching contact molecules for a query compound

**Fig. 7:**
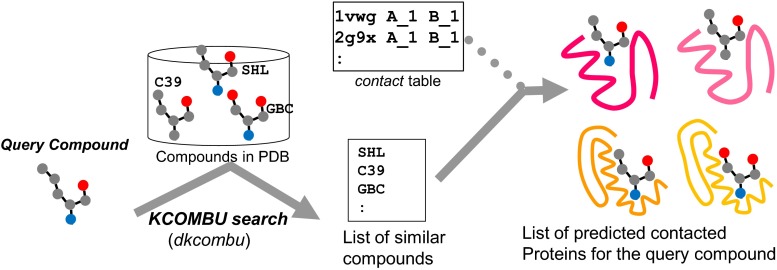
Overview of the service “searching contact proteins for the query compound”

**Fig. 8:**
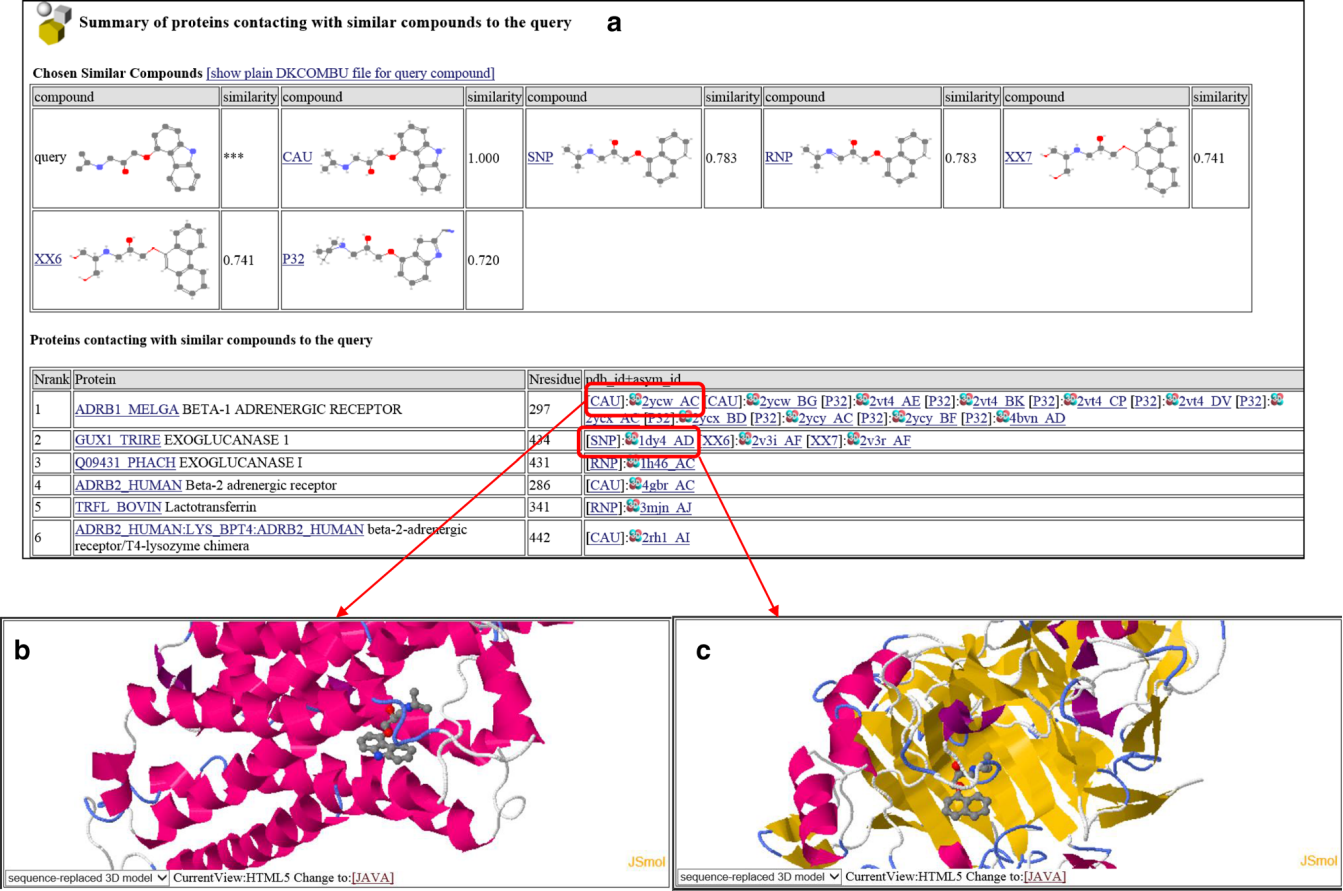
Snapshots of the service “searching contact proteins for the query compound”. This is the search result for the query compound carazolol (PDB three-letter code: CAU). **a** A list of similar compound to the query and a list of proteins contacting the similar compounds to the query. **b** A complex 3D structure of beta-1 adrenergic receptor and carazolol (CAU), taken from PDBcode 2ycw. **c** A complex 3D structure of exoglucanase 1 and S-propranolol (SNP) taken from PDBcode 1dy4

For a query small chemical compound, the HOMCOS server also searches contacting proteins. A user can specify a query 2D chemical structure by various methods: three-letter PDB code, SMILES string [51], and uploading a chemical structure file (SDF, MOL2, PDB). The computation procedure is described in Fig. 7. The server performs a 2D similarity search with the given query chemical compound for the database of all of the chemical compounds appearing in the PDB, using the *dkombu* program [40, 41]. The *dkcombu* program employs the combination of the atom pair descriptor search [52] and the 2D maximum common substructure search [40, 41]. The database of compounds contains all of the molecules with 3-letter codes (comp_id). Contacting proteins with these similar compounds are obtained by searching the *contact* table, and they are regarded as predicted contacting proteins with the query compound. Figure 8 shows an example of this service, using carazolol (comp_id: CAU) as the query compound. Carazolol is a partial inverse agonist of the beta adrenergic receptor. The HOMCOS server searched six similar compounds with Tanimoto similarity >0.7, and provided the list of proteins binding to one of these six compounds (Fig. 8a). As we expected, the beta-1 and beta-2 adrenergic receptors were included in the list. Surprisingly, two enzymes, exoglucanase 1 and lactotransferrin, were found in the list. Figure 8b shows the 3D structures of beta-1 adrenergic receptor with carazolol (CAU), and Fig. 8c shows exoglucanase 1 with S-propranolol (SNP), which is similar to CAU with Tanimoto index = 0.783. From this result, we can hypothesize that the carazolol may bind to exoglucanase 1 and lactotransferrin, which may lead to unexpected side effects. Of course, we have to realize that this is just a hypothesis. These predictions are based on the similar property principle, which states that “similar molecules have similar common binding properties”, but this rule is just empirical. Users have to be careful when using these predictions, and especially when using weak similarities.

### Modeling complex 3D structure of a hetero protein multimer

**Fig. 9:**
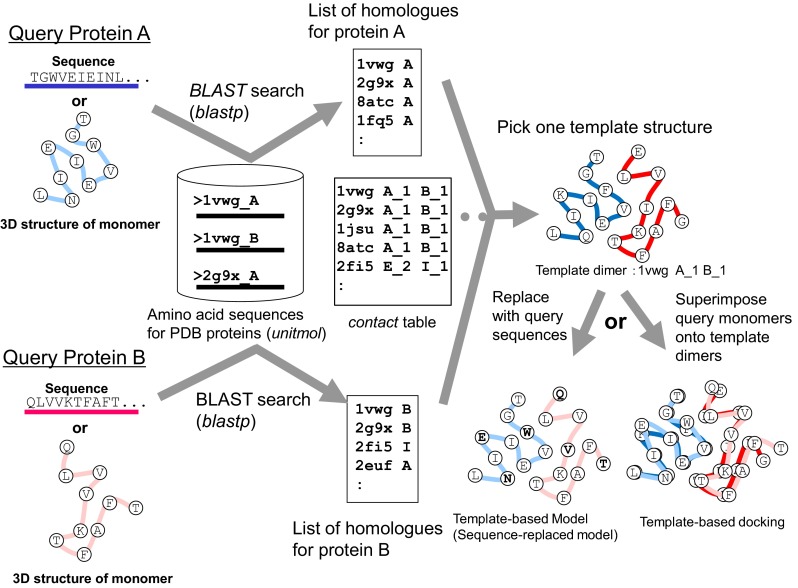
Overview of the service “modeling hetero protein multimer”

**Fig. 10:**
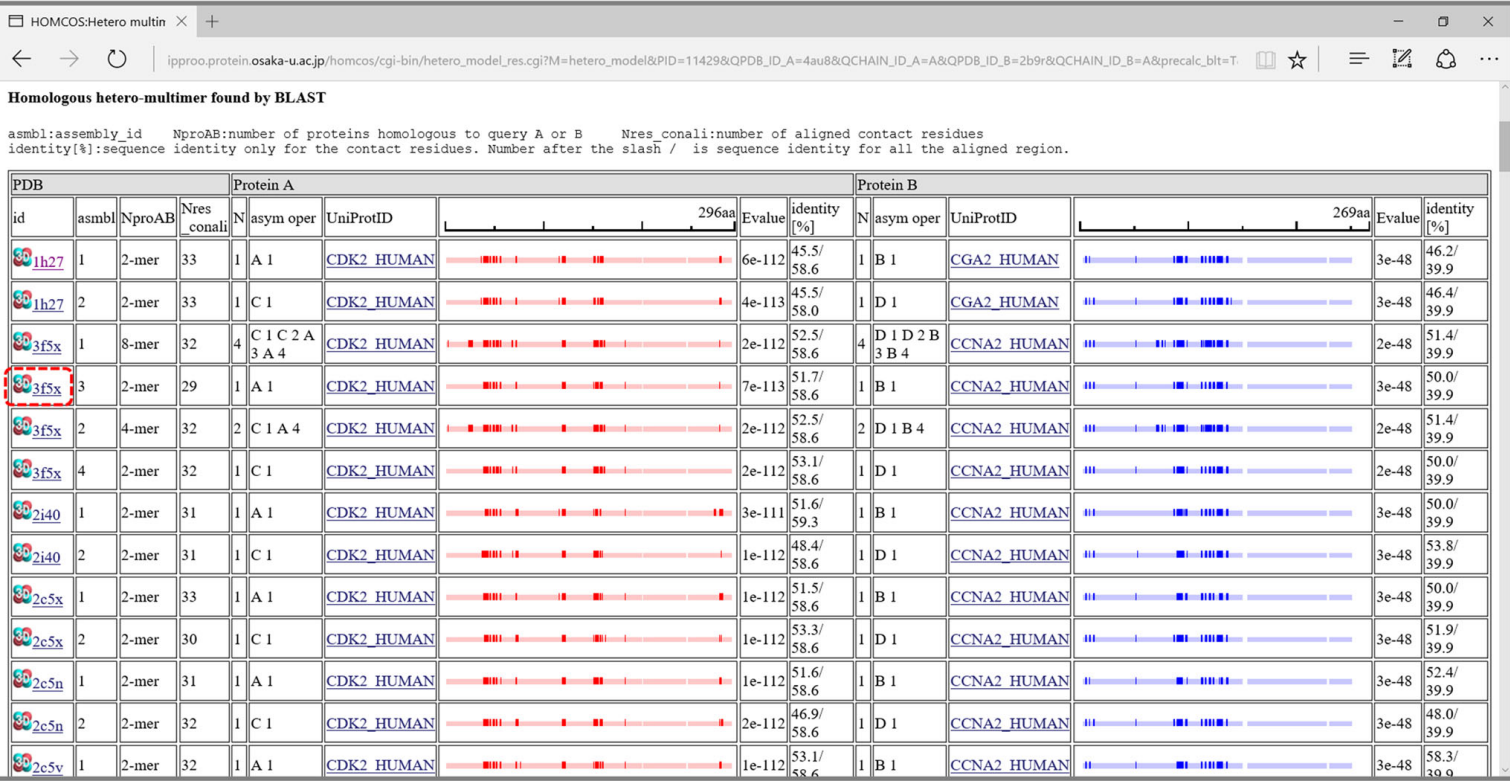
A snapshot of the bar view of “modeling hetero protein multimer”. This page summarizes results of two *blastp* searches for the two given query proteins 4au8 chain A (human cyclin-dependent kinase 5; CDK5) and 2b9r chain A (human cyclin B1). Pale *blue bars* are aligned regions for the query protein A (CDK5), and pale *red bars* are those for the query protein B (cyclin B1). Deep *blue* and *red boxes* show contacting residues. Instead of the two query structures, the server can accept two query sequences, such as CDK5_HUMAN and CCNB1_HUMAN

This service is for modeling the complex 3D structures of hetero multimers from two query proteins. The computation procedure is described in Fig. 9. A user can specify a single query protein by various methods: an amino acid sequence, UniProt ID, PDB_ID + CHAIN_ID, and uploading a PDB file. The server performs two *blastp* searches [39] with the two given query protein sequences for all of the *unitmol* sequences in the PDB, to make two lists of the homologous proteins (*unitmol*). The server then checks if a 3D dimeric structure of the homologues exists, in which one of the proteins is homologous to one query protein, and another protein is homologous to the other query protein. If the homologous multimers are found, then they can be used as the template for complex modeling. In contrast to the previous version of HOMCOS, our new server can model not only dimeric structures, but also multimeric structures composed of two different proteins, such as the tetrameric structures of hemoglobin alpha and beta chains. Figure 10 shows the search results for the two given query protein structures, 4au8 chain A (human cyclin-dependent kinase 5; CDK5) and 2b9r chain A (human cyclin B1). For these two proteins, we found several homologous complexes, such as CDK2 and CGA2.

### 3D model viewer

**Fig. 11:**
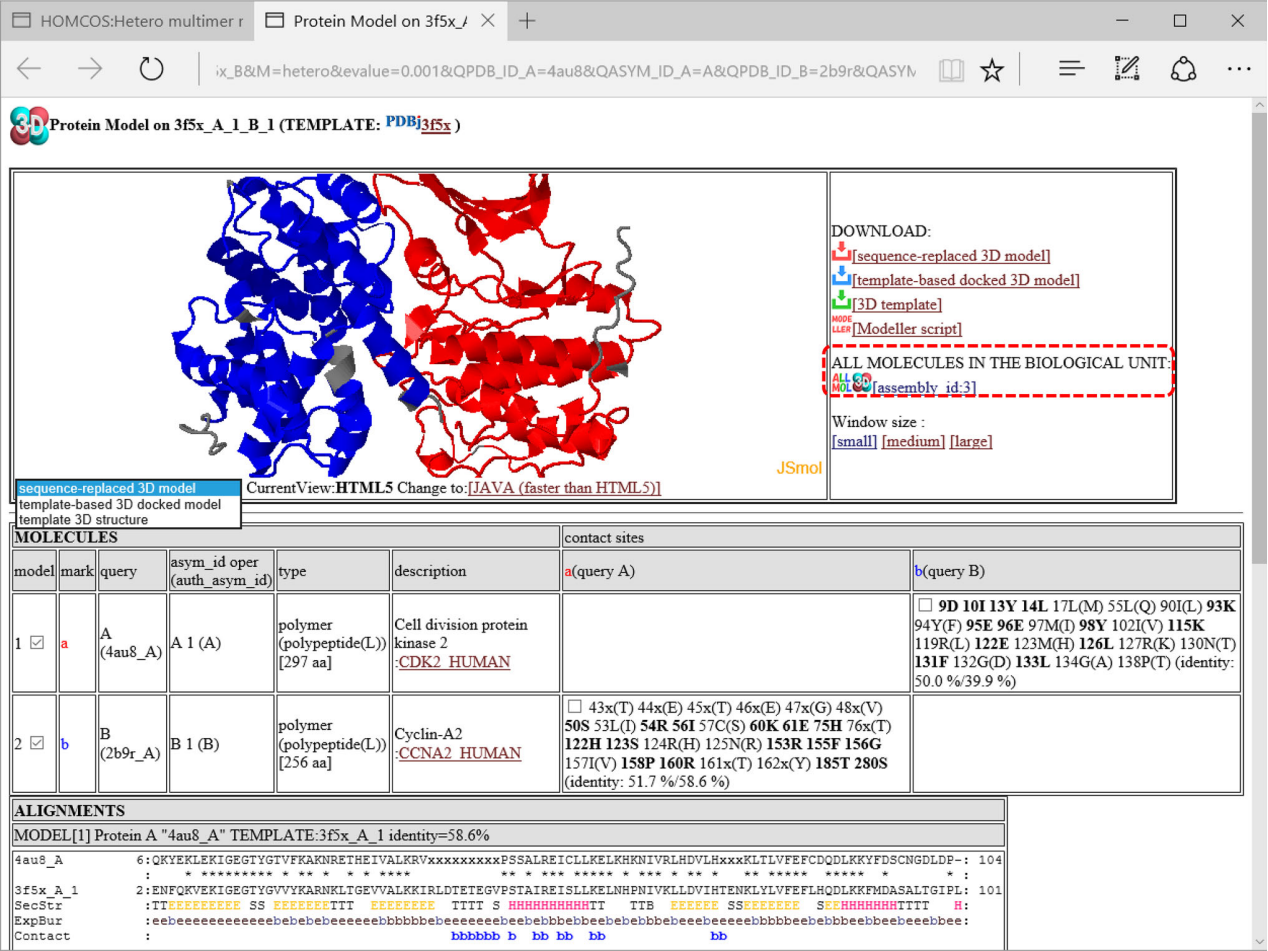
A snapshot of the window for 3D model. This page shows a 3D model of the complex of CDK5 (PDBcode:4au8 chainA) and cyclin B1(PDBcode:2b9r chain A) modeled by the template complex of CDK2 and cyclin A2 (PDBcode:1h27). The 3D structure of the sequence-replaced model is shown at the *top left* of the page using JSmol or Jmol. This model is created simply by replacing the residue name and number with those of the query protein according to the BLAST alignment. Non-aligned regions of the template structure are displayed in *gray color*. At the *top right* of the page, there are several links for downloading models are placed: downloading sequence-replaced model, template-based docked 3D model, template, and MODELLER script [53]. The link “ALL MOLECULES IN THE BIOLOGICAL UNIT” generates a new page including all of the molecules in one of the biological units (shown in Fig. 12). At the *middle* of the page, all of the template molecules in this model are shown. Residue numbers and residue names of the contact sites of the query proteins are shown in the left column (“contact sites”). If the site has the common amino acid for the query and the template, the site is shown in *bold*. At the end of the column “contact sites”, two sequence identities are shown, such as “50.0/39.9” and “51.7/58.6”. The first number is the sequence identity of the contact site, and the second number is the identity of all the aligned sites. The identity of the contact site is a good measure for the quality of the model, especially for the small chemical compound. At the bottom of the page, alignments between the query and the template proteins are shown. Contact sites are indicated by the “mark” *letters*, such as “*b*”

We use the common page for 3D model viewing for our five HOMCOS services. As an example, we will explain the case of the 3D model of the hetero protein multimer. On the results page of the hetero protein multimer in Fig. 10, if a user clicks the “3D” icon for each template complex, the window for the 3D model appears (Fig. 11). A sequence-replaced model is immediately generated by the server, and shown by JSmol or Jmol (http://www.jmol.org). This model is created simply by replacing the residue names and numbers with those of the query protein according to the BLAST alignment. The model can be generated with much less computation time than a full atomic modeling, and it is sufficiently useful for observing its molecular geometry at residue-level resolution. Of course, it cannot be used for docking and molecular dynamics simulation, because its substituted side chains are not correctly modeled and all of the inserted residues are missed. For users who desire more detailed models, the HOMCOS server provides a script file for the MODELLER program [53], which can be generated by clicking the icon at the top left of the windows (Fig. 11). Using the downloaded script file and template PDB files, users can immediately start the modeling calculation, if they already have the MODELLER program installed in their computer. The template-based 3D docked model can also be generated immediately, if the PDB IDs or uploaded PDB files for query proteins are assigned. It is built by assembling several 3D structures of query proteins using the RMSD fitting of the query residues on the corresponding residues of the template 3D structures of the complex. This modeling is useful if the precise monomeric 3D structure of the query protein is available, because the monomeric conformation of each subunit 3D structure is conserved during the modeling. The disadvantage of this modeling is that atomic crashes are often observed at the interfaces of the subunits, because any conformational changes occurring upon association cannot be considered.

**Fig. 12:**
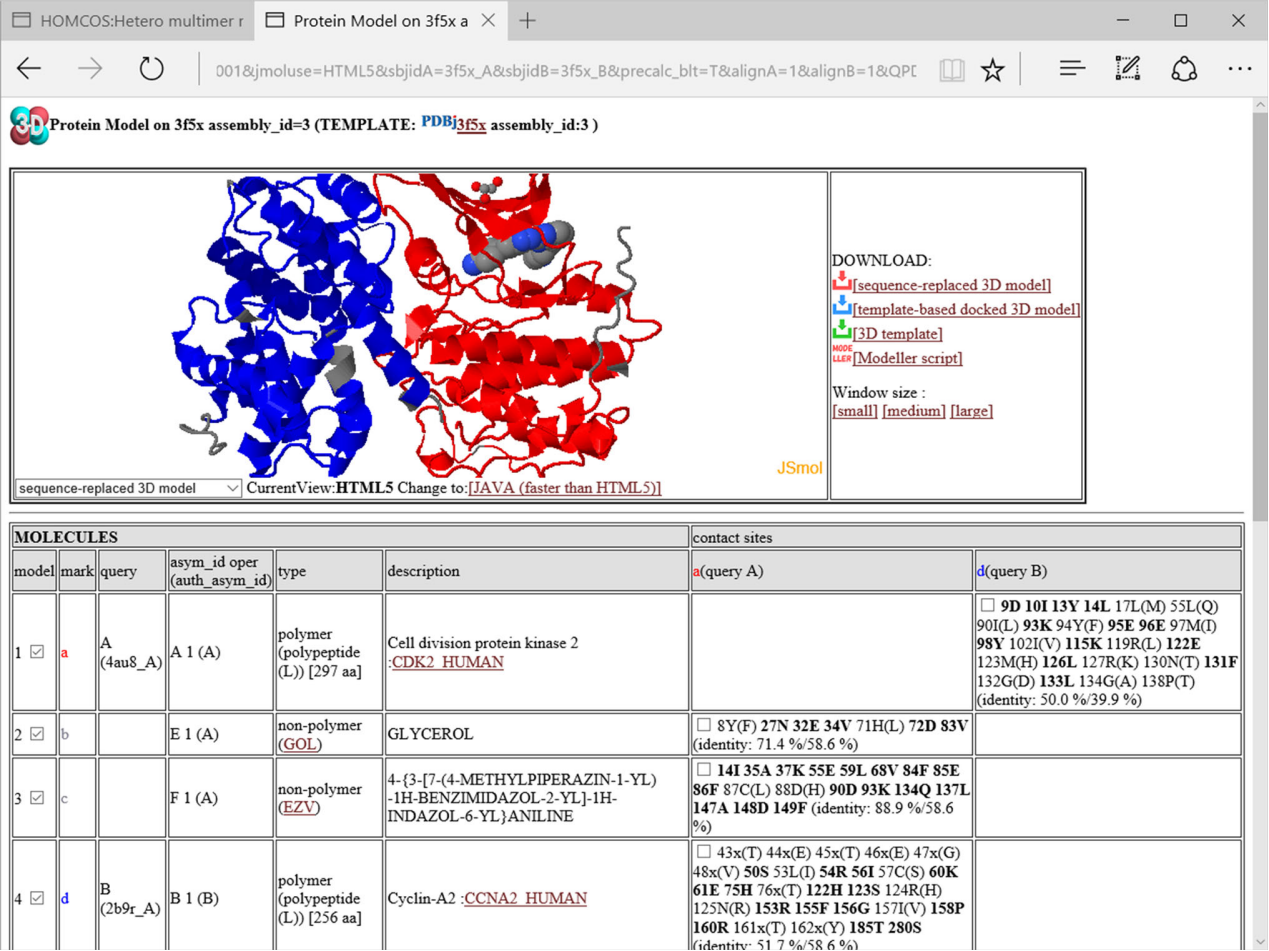
A snapshot of the window for the 3D model using all of the molecules in the biological unit. This page is shown by clicking the link “ALL MOLECULES IN THE BIOLOGICAL UNIT” in Fig. 11. It contains the molecules in PDBcode:3f5x with assembly_id = 3. Two additional molecules are added: GOL and EZV

In the model linked directly from the hetero oligomer page, only two protein molecules are always included (Fig. 11). However, other molecules are often included in the biological unit, and they may be useful for discussing the function of the modeled structure. For this purpose, the link “ALL MOLECULES IN THE BIOLOGICAL UNIT” is available at the top right of the 3D model page (Fig. 11). If this link is clicked, then the page is reloaded to include all of the molecules in the biological unit with the specific assembly_id. Figure 12 shows all of the molecules in PDBcode:3f5x with assembly_id = 3. In addition to the CDK2 and CCNA2 proteins, two additional molecules, GOL and EZV are present. As the molecule EZV is an inhibitor of ATP, the binding position of EZV should be similar to that of ATP, which is necessary for the protein kinase activity. Therefore, the model of the four molecules has more functional information than the model with just two proteins. If a user clicks the links for downloading models (such as the sequence replaced model, template-based docked 3D model, and MODELLER script), then a generated model will include these additional template molecules without any transformation of their poses and conformations. In general, template molecules without any assignments of query molecules, are included within a 3D model without any transformation. These molecules have a blank box in the third column “query”, in the table shown at the middle of the 3D model view. From the service “searching contact molecules for a query protein” explained in the previous section, molecules without any query assignment always appear in the 3D model views. In this service, only one protein molecule is modeled by replacement with the query sequence, and all other molecules are used without modifications.

### Modeling complex 3D structure of a protein-compound complex

**Fig. 13:**
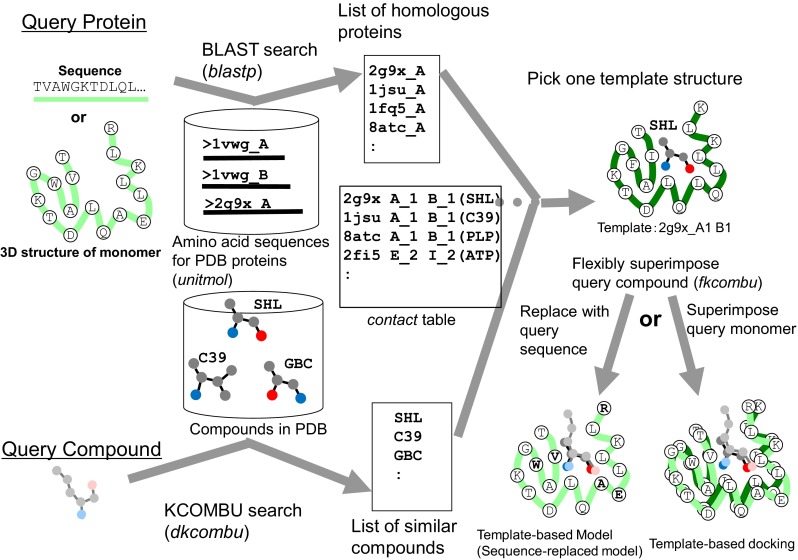
Overview of the service “modeling protein-compound complex”

**Fig. 14:**
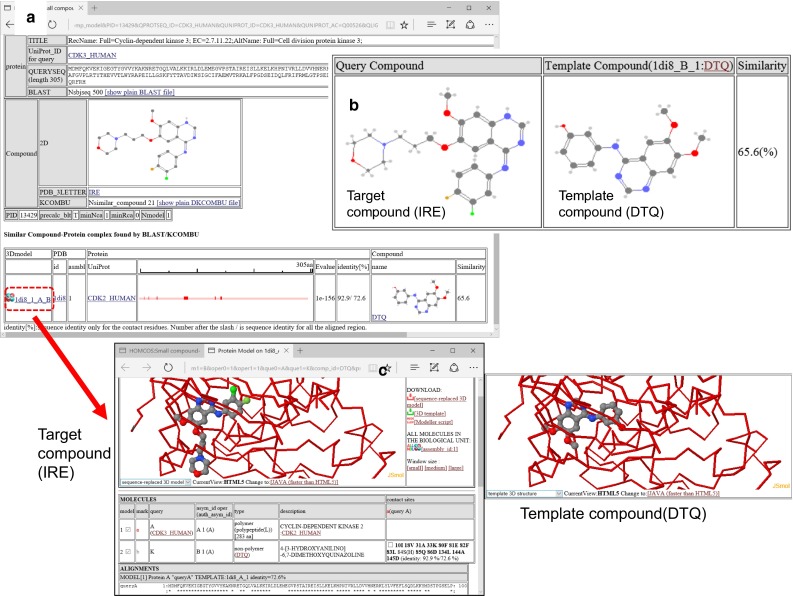
Snapshots of the “compound-protein complex”. These pages shows the result of modeling using the amino acid sequence of CDK3_HUMAN and the three-letter code IRE (iressa) as the queries. **a** A summary of the BLAST search and the KCOMBU search. A list of template structures is shown containing a homologue of the query protein and a similar compound to the query compound is shown. **b** Chemical structure comparison between the target query compound IRE and the template compound DTQ. **c** 3D model view based on the template structure 1di8. Both the 3D model with IRE and the template structure with DTQ are shown

This service is for modeling the complex 3D structure of one protein and one small chemical compound. The computation procedure is described in Fig. 13, and an example of the service on the Web page is shown in Fig. 14. A user inputs a query protein as its amino acid sequence or its 3D structures, as with the other services require. In addition, a query chemical structure is input by various methods: three-letter code of PDB, SMILES string [51], and uploading a chemical structure file (SDF, MOL2, PDB). For the 3D modeling, the uploaded chemical structure file should not be 2D, but should have a 3D conformation with sufficiently quality for the initial conformation. The server performs a *blastp* search [39] with one given query protein sequence for all of the *unitmol* sequences in the PDB, to make a list of the homologous proteins (*unitmol*). The server also performs a chemical similarity search with the given query chemical compound for all of the chemical compounds appearing in the PDB, by the *dkcombu* program [40, 41]. The server then checks if the 3D structure of a similar compound-protein complex exists, in which the protein is homologous to the query protein, and the compound is similar to the query compound. If the similar 3D complexes are found, then they can be used as the template for complex modeling (Fig. 14a). The conformation of the query protein is modelled in the same way to the previous subsection: simple sequence-replacing model or template-based docked 3D model. The conformation of the query compound is modelled by our flexible superposition program *fkcombu* [30]. The program *fkcombu* aligns the query target compound based on the atomic correspondences with the template compound, by changing the conformation of the target. The atomic correspondences are calculated by the maximum common substructure algorithm [40], as shown in Fig. 14b. The model and the template 3D structures are shown in Fig. 14c. The prediction accuracy of a compound 3D structure depends on the chemical similarities between the query and template compounds. We reported that if the target and template compounds have a Tanimoto coefficient >70 %, then the expected RMSD of the 3D conformation is <2.0 Å [30]. Users have to be careful when using predictions based on a template with <70 % similarities.

### Relationship to VaProS server

Some of the services of the HOMCOS server can be used through the VaProS server (http://p4d-infor.nig.ac.jp/vapros/). The VaProS is an integrated database system for structural life science, it includes not only the 3D structural database, but also the databases of sequences, molecular interactions, expressions, and diseases. It stands for “Variation effect on PROTein Structure and function”, because one of the important goals of the VaProS system is analyzing phenotypic effects of mutation using protein 3D structures. The page of the service “contact molecules for query protein” is shown in the VaProS page, with the name “3D Interaction”. For fast searching from the VaProS, the HOMCOS server stores pre-calculated BLAST results for all of the human sequences stored in UniProt. The VaProS server has the “Molecular Interaction” view, which shows molecular interactions as nodes and edges. At each edge for a protein–protein interaction, a link is placed to the service “modeling complex 3D structure of hetero protein multimer”.

### Comparison with other servers

As we explained in the introduction, there are many servers for protein–protein interactions (PPIs) [7, 8, 10–12, 22, 23, 28]. The clear advantage of HOMCOS over these PPI servers is that it can deal with all of the molecules stored in the PDB. As shown in Figs. 5 and 6, the summaries considering all of the contacting molecules provide comprehensive insight about the target protein. The 3D structures of two proteins and a chemical compound often provide more functional information, as shown in Fig. 12. This extension is enabled largely by the mmCIF format files of the 3D structures. Especially, the identifier “asym_id” and the clear descriptions of the biological units are indispensable for our server, but are not included in the classical PDB format. Our program KCOMBU also contributes to the searching and superimposition of the chemical compound structures.

Another advantage of the HOMCOS server is its fast computation. Some WEB servers take more than half an hour for calculations [11, 23], or provide only pre-calculated 3D models [8, 38]. In contrast, the HOMCOS server only takes 1–3 min for searching and modeling for any user-input query molecules. This is simply because the HOMCOS server employs fast searching programs (*blastp* and *dkcombu*), and provides only sequence-replaced models as a default, and makes time-consuming atomic modeling optional. We think that our strategy provides a good balance between fast response and precise prediction for template-based modeling. We expect that users would always like to quickly know whether templates are available for their target molecules, because template-based modeling is useless when templates are not found. Detailed predictions will only be required when the user performs more detailed analyses, such as molecular simulations.

## Discussion

### Further improvement of the server

The current HOMCOS server has many more functions than our previous server, established in 2008. However, the server still has room for improvement. First of all, we only employ the program *blastp* for protein sequence searches, but more sensitive profile-based programs or structure comparison programs are sometimes required to detect remote homologues. They can increase the coverage of the model at the expenses of the precision, and can provide longer and more accurate alignments with templates. The technical problem in implementing them in our server is their larger computation time. We first plan to include PSI-BLAST [39] in the near future, considering the balance between its computation cost and advantages.

Second, quality evaluations of the model should be introduced. Currently, the HOMCOS server only shows the sequence similarity and chemical similarity between the target query and template molecules. These similarities are reported to be good measures for the quality of models [30, 54], and detailed analyses for the evolutions of complex 3D structures will be reported elsewhere. Our analyses show that the sequence identity of the contact sites has a better correlation with the structural difference of the complex, especially for the complex with small chemical compounds. The HOMCOS server shows the new measure, the sequence identity of the contact sites, in the “Summary Bar” view (Fig. 5) and the 3D model view (Fig. 11). Other measures, such as interfacial contact potential energies may need to be introduced.

Third, some molecules such as nucleotides and polysaccharides cannot be searched and modeled by the current HOMCOS server, although our database contains all of the molecules in the PDB. As shown in Fig. 5, the service “searching contact molecules for query protein” can find the complex structures with nucleotides and polysaccharides. If their sequences and chemical structures do not have to be changed, they can be used as the rigid body template molecule with the homologous template protein. We hope this function is quite useful, but may not be sufficient. There are no standard tools for searching polysaccharides, and for modifying chemical structures of nucleotides and polysaccharides, although a nucleotide sequence can be searched by the *blastn* program [39]. We may have to develop these tools, if many users request the modeling of these molecules.

Fourth, we maximally accept two query protein sequences and one chemical query compound; however, more query molecules may have to be accepted. In principle, the modeling of multiple query sequences is not impossible. The problems are rather technical: its high computation cost and the need for a user-interface to input many query molecules.

Finally, our predictions of binding molecules have limited accuracies. They are performed by our two services, “searching contacting molecules for a query proteins” and “searching contacting proteins for a query compound”. These predictions are based on the similar property principle, which states that “similar molecules have similar common binding properties”. However, this rule is simply empirical, and exceptional cases cannot be ignored. Fukuhara et al. [9] reported that sequence similarity is essential to predict interactions, however, its accuracy is limited. Users have to be careful when using these services, especially when similarities are weak. More studies must be performed for more precise prediction of molecular interactions.

## Concluding remarks

Our new server has been greatly improved in comparison to its previous version, established in 2008. It contains all of the different types of molecules in the PDB, and provide a useful summary of the contact molecules for a query protein. We hope our server will be useful for a wide range of life-science researchers, and will contribute toward making the full use of the 3D structural data stored in the PDB.
